# On the Deep Learning Models for EEG-Based Brain-Computer Interface Using Motor Imagery

**DOI:** 10.1109/TNSRE.2022.3198041

**Published:** 2022-08-19

**Authors:** Hao Zhu, Dylan Forenzo, Bin He

**Affiliations:** Department of Biomedical Engineering, Carnegie Mellon University, Pittsburgh, PA 15213 USA

**Keywords:** Brain-computer interface, BCI, deep learning, EEG, motor imagery

## Abstract

Motor imagery (MI) based brain-computer interface (BCI) is an important BCI paradigm which requires powerful classifiers. Recent development of deep learning technology has prompted considerable interest in using deep learning for classification and resulted in multiple models. Finding the best performing models among them would be beneficial for designing better BCI systems and classifiers going forward. However, it is difficult to directly compare performance of various models through the original publications, since the datasets used to test the models are different from each other, too small, or even not publicly available. In this work, we selected five MI-EEG deep classification models proposed recently: EEGNet, Shallow & Deep ConvNet, MB3D and ParaAtt, and tested them on two large, publicly available, databases with 42 and 62 human subjects. Our results show that the models performed similarly on one dataset while EEGNet performed the best on the second with a relatively small training cost using the parameters that we evaluated.

## I. Introduction

Brain-computer interface (BCI) is an emerging technology which can measure brain activity and convert it into artificial outputs that can replace, restore, enhance, supplement, or improve natural CNS outputs [1]. Among different BCI tasks, motor imagery (MI) is one of the commonly used paradigms [2], [3], [4], [5], [6], and is typically defined as imagining the movement of a body part without actual motor execution. This has been shown to share a similar mechanism as real motor execution [7], [8], and enabled human subjects to control a computer cursor [2], drone [9], and robotic arm [6], [10]. It has shown potential in helping patients with motor disabilities interact with the environment by controlling devices such as computer cursors, prostheses, and wheelchairs [11]. Electroencephalography (EEG) based BCI is one type of commonly used BCI techniques due to its non-invasive nature that does not require any surgical procedure for use. One of the main components of these BCI systems is the classification of circumscribed and transient EEG changes like event-related synchronization (ERS) or event-related desynchronization (ERD) during different types of motor imagery [3]. Developing a robust decoding algorithm is one of the important things in improving BCI research and user experiences [12]. There are lots of successful feature extraction methods, such as common spatial patterns (CSP) methods [13], [14]. Among the CSP methods, filter bank common spatial pattern (FBCSP) [15] is one of the most popular algorithms which uses a group of band-pass filters to extract the optimal spatial features, and has achieved robust performance in MI classification tasks. However, this kind of traditional framework requires that the process of feature extraction/selection and feature classification are separated, which requires manual work and prior knowledge by the operator, which could lead to bias and inefficiencies.

Recently, with the rapid development of high-powered computing devices, deep learning has become increasingly popular in many fields. A major advantage of bringing in deep learning technology into BCI systems is that feature extraction and classification steps can be jointly learned directly from data, also known as ‘end-to-end’ learning [16]. Several groups have been working on deep neural networks for MI classification [17] and published several models. Schirrmeister *et al*. [18] explored deep neural network structures for MI classification. They presented four different models: shallow CNN, deep CNN, hybrid shallow+deep CNN and a residual network and showed that their shallow CNN and deep CNN substantially outperformed the traditional method while the other two did not. Sakhavi *et al*. reported an envelope representation for EEG-based motor imagery classification and combined it with a 5-layer convolutional neural network, which increased the classification accuracy by 7% on BCI competition dataset IV [19]. EEGNet [20] is another successful network which combined different basic convolutional layers together and achieved good performance on multiple datasets. Several other CNN-based structures have also been explored in motor imagery classification, showing that it is a popular architecture choice [21], [22], [23]. In addition, other studies have tried to combine traditional CNN layers with other tools to improve performance, such as self-attention modules [24] or stacked autoencoders [25]. Amin *et al*. designed a fusion model, which fuses multiple CNN networks to extract different levels of feature characteristics, and achieved substantial improvements of classification accuracy on multiple datasets [26].

A common approach to using EEG signals as inputs to these models is to represent the signal as a two-dimensional (2D) array where each row is a timeseries recording of a single EEG channel, and each column represents signal from different channels at a time. This method is convenient for visualizing the EEG signal and mimics the 2D structure of images in image processing where CNNs have been used extensively. However, this representation loses the spatial relationship among channels in the EEG montage, which may contain information useful for MI classification. To deal with this problem, some studies have explored a 3D representation of EEG data instead [27], [28].

The variety of deep learning architectures proposed in the recent publications shows the field’s effort to improve MI-EEG classification by using new tools. Comparing the performance of these architectures offers a strategy for improving future BCI classifiers by further exploring the models and tools that lead to higher accuracies. However, a major difficulty in comparing the proposed architectures is that the datasets used to train and test these models are usually different between studies, or may not even be publicly available. The differences in the datasets may account for some of the differences in published accuracies, where some models may perform better on some datasets over others. In addition, the most commonly used motor imagery dataset is the BCI competition dataset IV [29], which includes only 9 subjects and does not include feedback. The small number of subjects could lead to a large variance in evaluation because the generalizability of a model may be questioned. This will lead to a strong need of comparing existing models on a larger, publicly available database, which will help in selecting deep learning models and design choices to build better MI classifiers in the future. In this study, we selected 5 MI-EEG deep learning models with released code from 4 recent studies: EEGNet [20], Deep & shallow ConvNet [18], Multi-branch 3D CNN [28] and parallel self-attention network [24]. We designed a group of experiments to test these models on two large publically available motor imagery databases: MBT-42 [30] with 42 human subjects and Med-62 [31], [32] with 62 human subjects, each of which contains multiple BCI sessions with online feedbacks. We evaluated each model both on its classification accuracy of L/R (left/right) motor imagery tasks and on its model training cost. Our results are as follows: Among the five models we selected, all of the models performed similarly on the first dataset, while EEGNet performed the best on the second in terms of classification accuracy and training time for the hyperparameters and training choices we tested. We also show that a simple preprocessing step is important to deep learning model training, and its magnitude of improvement depends on the dataset.

## II. Material and Methods

### A. Datasets

Two large, publicly available datasets were used in this study to compare the performances of several recently published deep learning neural networks for MI classification. More information on these datasets is available in the original publications [30], [31].

#### 1) MBT-42:

**Fig. 1.**
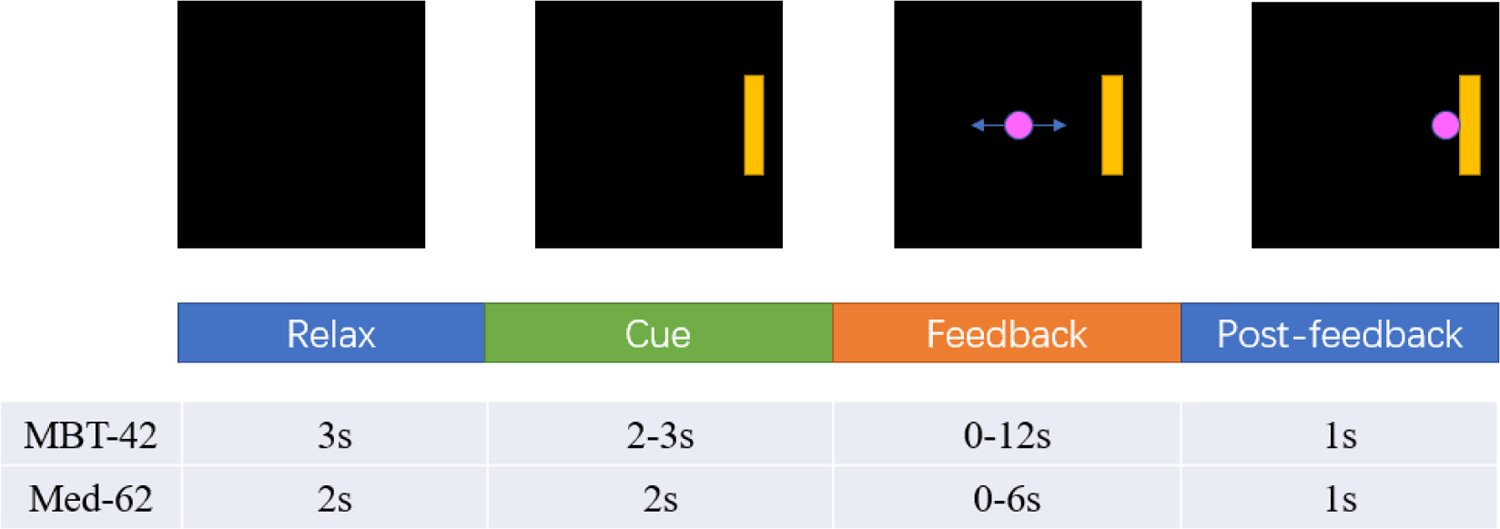
Trial structure of two datasets. A trial starts from a relax stage, shown as the blank screen. Then a rectangle target will appear at either side of the screen, giving subject the hint of direction to perform motor imagery. At feedback stage, a circle cursor will appear on the center of the screen and will move towards either side based on motor imagery of the subject. After the cursor reach or miss the target, or the time exceeds the limit, the cursor will be frozen at the post-feedback stage. The length of each stage is summarized in the table.

This dataset was recorded during a series of MI-BCI training [30]. A total number of 42 healthy human subjects took part in this L/R cursor moving task. Here, the subjects’ goal is to perform left/right motor imagery and to direct the cursor to reach the target, which appears randomly at either side of the screen (Figure 1). The complete dataset consists of three experiments involving 16, 12, and 14 subjects respectively. All the subjects participated in three sessions of online control tasks. In the first experiment, there are 125 trials within each session. In the second and third experiment, there are 120 trials. The EEG signals are recorded using a 62 channel Neuroscan system in the first two experiments, and a 64 channel Biosemi Active Two system in the third experiment. The trial structures of the three experiments are similar, each including 4 stages. A trial will start with a relaxation stage, which is considered as the rest time between two trials. Next is the cue stage, where two square targets are placed on the left and right of the screen. One of these targets is made visible as a yellow square to provide a goal for the subject. The left and right trials are randomly distributed and balanced within sessions. After that, the feedback stage will start. A round pink cursor appears and starts moving based on the control signal provided by the classifier. This will give subjects the feedback about the effects of their motor imagery performance. If the cursor reaches the visible target the trial ends and results in a “hit”. If the cursor reaches the invisible target on the opposite side of the screen the trial also ends and results in a “miss”. If neither target is hit before the time limit, the trial ends and results in an “abort” trial. The last stage is the post-feedback stage, where the cursor will be frozen for one second. Subjects are only explicitly instructed to perform motor imagery during the feedback stage, though they may opt to perform it during other stages as well. In the first two experiments, the EEG signals had gone through a bandpass filter between 0.5 and 200 Hz and a notch filter of 60 Hz, and was down-sampled to 100 Hz. In the third experiment, the EEG signals had gone through a bandpass filter between 0.16 and 100 Hz and a notch filter of 60 Hz, and was down-sampled to 128Hz.

MBT-42 dataset is openly available at the following URL/DOI: http://dx.doi.org/10.6084/m9.figshare.7959572.

#### 2) Med-62:

This dataset is collected to explore the effect of meditation on motor imagery performance [31], [32]. In this dataset, 62 subjects participated in cursor movement control tasks of three types: left/right (LR) movement only, up/down (UD) only, and combined 2D movement (2D). Each subject completed a total number of 7–11 sessions of online BCI tasks. Each session is comprised of 450 trials counting all of the three tasks, 150 trials for each task. The EEG data is recorded by a 64-channel EEG Neuroscan cap. The data are sampled at 1,000 Hz and have already been bandpass-filtered between 0.1 to 200 Hz, with a notch filter at 60 Hz as well. The trial structure is similar to the first dataset, including a relax stage, cue stage, feedback stage and post-feedback stage. The length of feedback stage varies from 0–6s depending on if and how quickly a target was hit. The online decoders of both datasets are similar, which including spatial filtering using Laplacian filter, estimating mu rhythm power by fitting an autoregressive model, and operating cursor movement based on lateralized mu rhythm power (C4-C3) for left/right movement tasks.

Here, we only use the first three sessions and only LR trials of this dataset to keep comparable between the two datasets. Med-62 dataset is openly available at the following URL/DOI: https://doi.org/10.6084/m9.figshare.13123148.

### B. Deep Learning Models

We chose to compare the following models which are specifically designed for motor imagery classification tasks and have released their codes for the models. This makes it easier to replicate the original authors’ work by testing their models in a new dataset. The released codes allow us to use the models most accurately as the way the authors intended. This framework can also be used in the future to compare more models as they are released. These models all adopt convolutional layers in their structures, which are widely used in processing temporal signals.

#### 1) EEGNet [20]:

EEGNet is a compact convolutional neural network combining depthwise and separable convolutions. It consists of 3 convolutional layers and 1 fully-connected layer, trying to encode several EEG feature extraction concepts like optimal spatial filtering and filter-bank construction.

#### 2) Deep & Shallow ConvNet [18]:

In this work, 4 different DNN structures, including shallow convolutional network, deep convolutional network, hybrid network and residual network, are explored and carefully compared to state-of-the-art methods. According to their results, shallow and deep ConvNet outperformed traditional FBCSP method while the other two did not, and we include both of shallow and deep network in our study.

#### 3) Multi-Branch 3D CNN [28]:

A typical deep learning model takes in a *C* × *T* matrix as input. In this situation, each channel will be treated equally and independently in the model, which will lose the spatial correlation among different channels. The novelty of this model is converting this 2D input into a 3D tensor, putting data from each channel into matrix entries arranged by its spatial position on the scalp. The channel arrangement in our experiment can be found in Supplementary Table S1. After that, the model uses 3D convolutional layers instead of 2D convolutional layers to generate its prediction.

#### 4) Parallel Self-Attention Network [24]:

ParaAtt introduced the popular self-attention concept in deep learning models [33] into EEG classification. Attention modules can automatically capture global relationships among input entries. With the parallel spatial-temporal self-attention mechanism, high-level distinguishable spatial-temporal features of raw signal data can be captured.

The detailed structures and parameters of these models we used in our experiment can be found in the supplementary Information.

### C. Data Analysis

In our study, we included both within-subject analysis and cross-subject analysis. Within-subject training will train a model specifically for each subject. For both datasets, we use the first two sessions as training set, and test on the third session. Within the training set, we split 20% of data samples for validation. For data pre-processing, following [24], we first perform exponential moving standardization to the raw data. For a signal vector **x** = *x*_1:*T*_, this standardization process can be formularized as:

$$d_{t}=x_{t}-\frac{\overset{t}{\underset{i=1}{\sum }}{\left(1-\alpha \right)}^{t-i}x_{i}}{\overset{t}{\underset{i=1}{\sum }}{\left(1-\alpha \right)}^{t-i}}$$

$$v_{t}=\frac{\overset{t}{\underset{i=1}{\sum }}{\left(1-\alpha \right)}^{t-i}d_{i}^{2}}{\overset{t}{\underset{i=1}{\sum }}{\left(1-\alpha \right)}^{t-i}}$$

$$x_{t}^{\prime }=\frac{d_{t}}{\text{max}\left(\sqrt{v_{t}},\epsilon \right)}$$

where *α* = 0.001 is the exponential factor, and *ϵ = *0001 is a small number to avoid division by zero. To keep the length of two datasets comparable, we then down-sampled Med-62 dataset to 100Hz. Since we can only assure that subjects are performing motor imagery during the feedback stage, we generate data samples from only the first 3 seconds of the feedback stage. Since the length of feedback stage in both datasets are variable, and some of them are shorter than 3 seconds, we pad the data samples with zeros if trial is too short. To compare the deep learning model performance with online performance and to ensure the fairness, we used only the first 3 seconds of feedback stage data to calculate the online accuracy. For each trial, if a decision was made before 3 seconds of feedback, then that decision is used as the result. Otherwise, if the trial lasts more than 3 seconds, then the target closer to the cursor at 3 seconds is taken as the result. The average length of feedback stage is: 5.16s in MBT-42 dataset and 5.74s in Med-62 dataset. The percentage of trials with feedback stage less than 3 seconds is: 21.4% in MBT-42 dataset and 5.9% in Med-62 dataset.

We performed cross-subject analysis on Med-62 dataset. Since MBT-42 dataset is recorded by different system for different subjects with different sampling frequency and number of channels, it was not included for cross-subjects analysis. We partitioned the data as follows: For each evaluation round, one out of 62 subjects was selected as the test set, and samples from all other 61 subjects are used for training and validation. Within the 61 subjects, training set and validation set are split at an 80:20 ratio. We trained each model using the training set, and select the best training epoch and parameters on the validation set. After that, we evaluated the trained model on the test subject and obtained an accuracy. This process was repeated for each of 62 subjects being used as the test subject. Similar to the within-subject analysis, we only used the data from the first three sessions for all subjects.

Full details of model and training parameters we used in our study can be found in the supplementary Information.

## III. Results

### A. Within-Subject Analysis

**Fig. 2.**
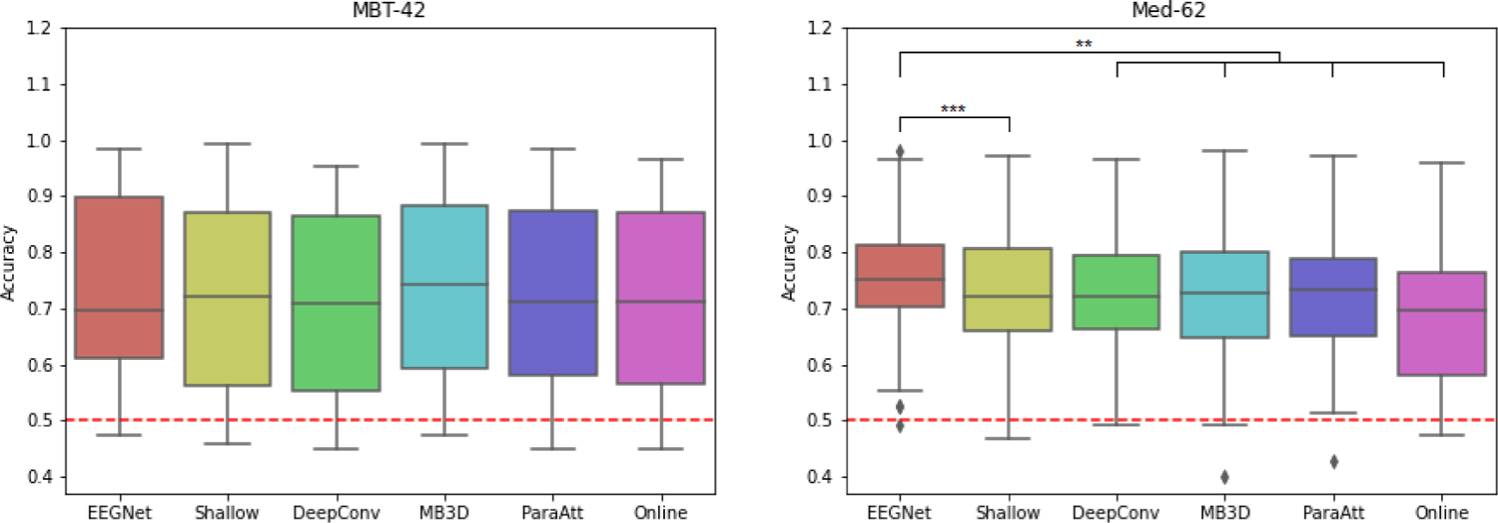
Box-plots of classification accuracies of deep learning models and online performance. Lower and upper box boundaries denote 25th and 75th percentiles, respectively. Lines inside box denote median. The “whiskers” extend to points that lie within 1.5 interquartile ranges (IQRs) of the lower and upper quartile, and then observations that fall outside this range are displayed independently. Red dashed lines denote the chance level. Stars denotes the statistically significant differences between model pairs (P values from Wilcoxon signed-rank test, **: P<0.01, ***:P<0.001). All P values have gone through adjustment of false-discovery-rate.

Under our analysis setting, all of the deep learning models achieved higher accuracies compared to the online accuracy in both datasets (Table I, Figure 2). The complete accuracy results can be found in Appendix. The Friedman test for repeated measurements show that there exist significant differences among the five models in both datasets (P<0.05 in MBT-42, P<0.001 in Med-62). In MBT-42, all of the models performed relatively similarly and no model significantly outperformed the others (P>0.05 on each pair, one-sided Wilcoxon signed-rank test, FDR adjusted). Note that although the difference of average accuracy between EEGNet and online experiment is larger (73.65% vs 70.90%), they perform similar under Wilcoxon test (P>0.2). In Med-62, EEGNet performed significantly better than each other models (P<0.001 compared to Shallow ConvNet, P<0.01 to others). All other model pairs do not have significant difference on performance. All of the P values have gone through adjustment of false-discovery-rate.

**Fig. 3.**
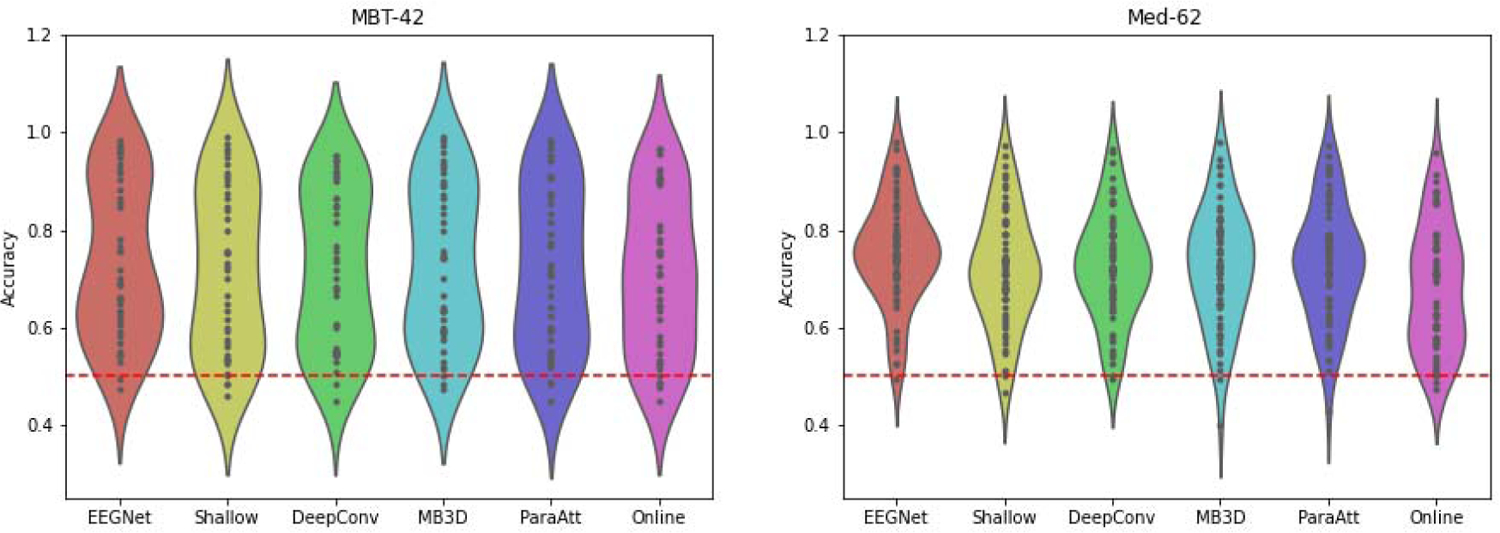
Distribution of model accuracies from different subjects. Each black point represents an accuracy from a single subject. The violin plot outlines illustrate the density of accuracies, i.e. the width of the colored area represents the proportion of subjects achieving accuracies at that level. Red dashed lines denote the chance level.

Figure 3 shows the distribution of model accuracies from different subjects. Each black point represents the model accuracy of one subject and the width of the colored area represents the density distribution of the accuracies. Here we can see a slight difference in the distributions between deep learning models and the online decoder, while the deep learning models are similar to each other.

### B. Effects of Preprocessing

**Fig. 4.**
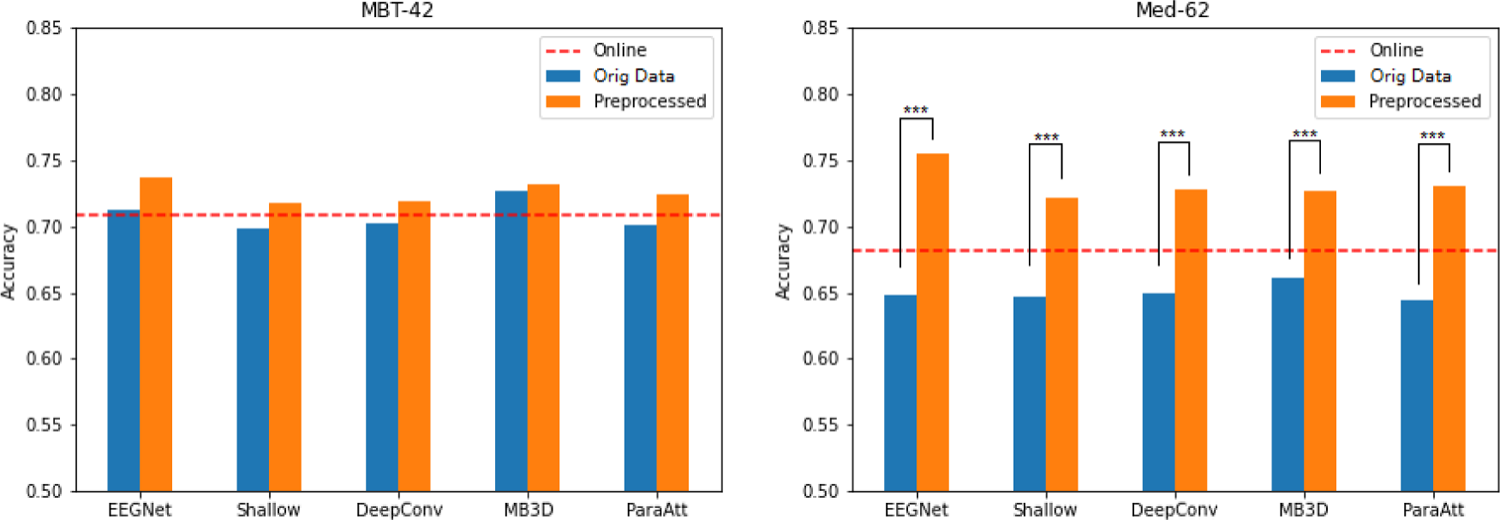
Comparison of models training on preprocessed data (using exponential moving standardization) and original data. Red dash lines mark the online accuracies. There is nearly no improvement in MBT-42 dataset, but significant improvement of all models in Med-62 dataset (P values from Wilcoxon signed-rank test, ***:P<0.001).

To explore the role of data preprocessing on model accuracy, we performed another group of experiments, which train deep learning models on the original data instead of preprocessed data using the same model structure. Figure 4 shows the comparison of the deep learning model classification accuracies training on preprocessed data and original data. We can see the preprocessing step has different effects on different datasets. On MBT-42 dataset, preprocessing only achieves limited improvement on deep learning model performance. None of the models shown have significant improvement when training on preprocessed data (P<0.05). However, on Med-62 dataset, each model achieved significant improvement by preprocessing (P<0.001). All of the P values have gone through adjustment of false-discovery-rate. In comparison with the online performance denoted by red dash line, we can see that most of the deep learning models training on original data cannot beat online performance, which indicates that simple steps of data preprocessing are necessary for the training of deep learning models under these conditions.

### C. Across-Subject Analysis

**Fig. 5.**
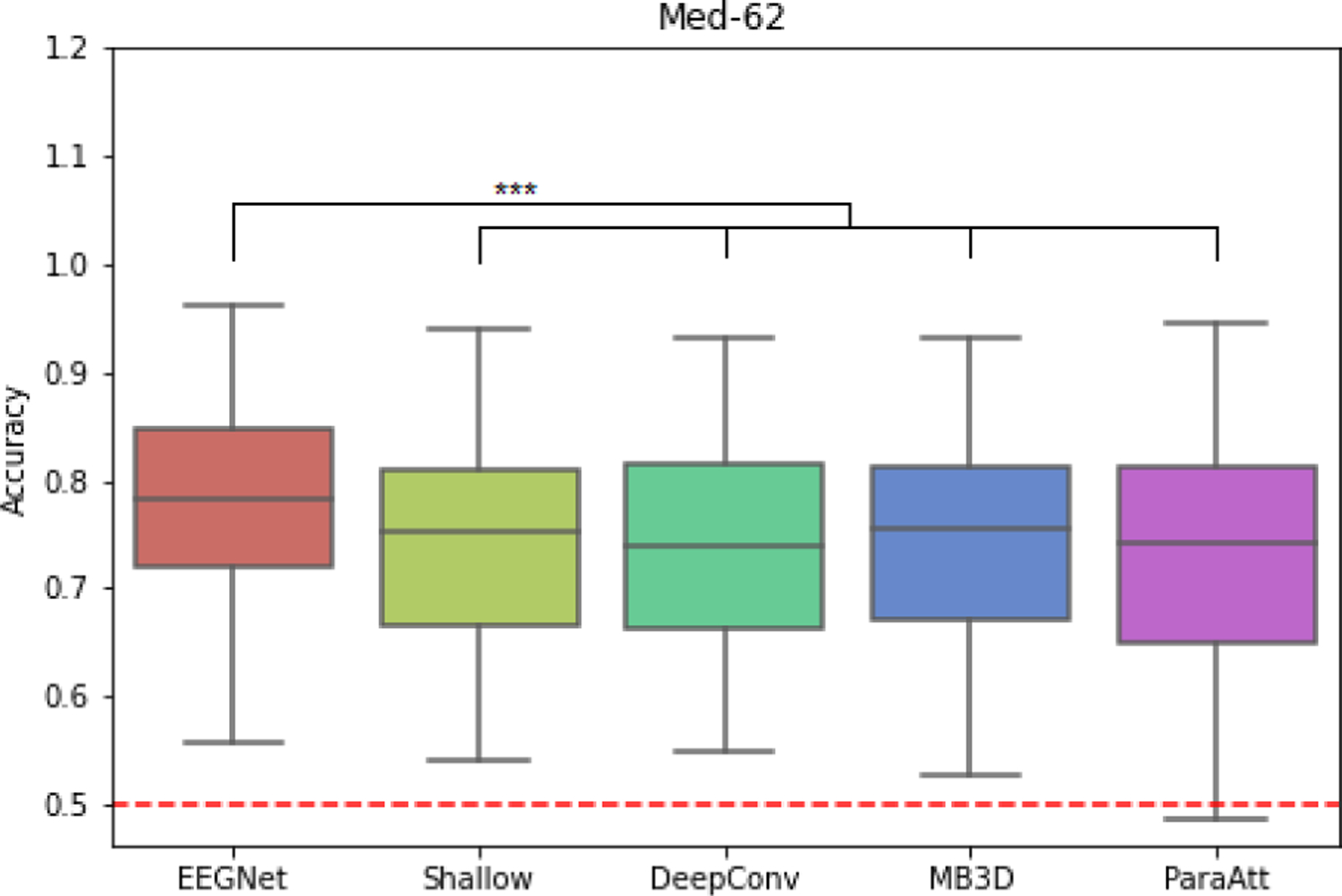
Box-plots of cross-subject classification accuracies of deep learning models over all subjects on Med-62 dataset. Lower and upper box boundaries denote 25th and 75th percentiles, respectively. Lines inside box denote median. The “whiskers” extend to points that lie within 1.5 interquartile ranges (IQRs) of the lower and upper quartile, and then observations that fall outside this range are displayed independently. Red dashed lines denote the chance level. Stars denotes the statistically significant differences between model pairs (P values from Wilcoxon signed-rank test, ***:P<0.001). All P values have gone through adjustment of false-discovery-rate.

The cross-subject classification results of different deep learning models on Med-62 dataset are shown in Figure 5. The Friedman test for repeated measurements show that there exist significant differences among the five models (P<0.001). EEGNet performed significantly better than each other models (P<0.001 to all other models, one-sided Wilcoxon signed-rank test, FDR adjusted). All other model pairs do not have significant difference on performance. All of the P values have gone through adjustment of false-discovery-rate.

### D. Computational Cost

Except from model accuracy, training cost is also an important criterion to evaluate a model. Table II compares the average training time of deep learning models on one subject. The timing tests are all operated on the BRIDGES-2 server at the Pittsburgh Supercomputing Center (PSC) [34] deployed with 40-thread Intel(R) Xeon(R) Gold 6248 CPU and one single core of Tesla V100 GPU. For EEGNet, Shallow & Deep ConvNet and ParaAtt, we trained 30 epoches for eacb subject, while for MB3D, we adjusted the epoch number to 15 since this model had a longer training time than the others. The result shows that MB3D’s unique structures of 3D convolution layers will lead to longer training time. Shallow & Deep ConvNet have the least training cost to achieve a fairly good performance.

We also compared model inference time of deep learning models. Inference time is the amount of time a trained model takes to generate an output from the input signal. The results are shown in Table III. We fixed all data input length to 300, and tested the model inference time on 125 samples and one single sample. Due to parallel computation, models can process faster on a batch of samples. Generally, the model inference time should be shorter than the update interval of real BCI systems, and 40ms is a commonly used value. All five models are able to decode one single sample shorter than this time, which means they all reserve the potential to put into real use.

## IV. Discussion

In this study, we have tested five previously reported BCI deep learning models on two large and publicly available left/right motor imagery classification datasets. There have been research testing deep learning algorithms on motor imagery tasks. Schirrmeister *et al*. [18] compared the performance of novel DL models against a traditional BCI classifier in an offline setting to show the potential benefits of using DL models for BCI decoding. In their work, ShallowConvNet and DeepConvNet achieved 85.3% and 84.0% accuracy respectively on two datasets, outperformed FBCSP baseline (82.1%). Lawhern *et al*. [20] compared EEGNet with ShallowConvNet, DeepConvNet and traditional approaches on both ERP and Oscillatory-based BCIs, and found that which model performs the best will change with datasets. MB3D network achieved 75.0% accuracy on BCI competition IV dataset in their original work [28]. Liu *et al*. [24] reported ParaAtt and compared it to multiple models. In their original work, ParaAtt achieved 78.5% accuracy on BCI competition IV dataset, which outperformed EEGNet (65.4%), DeepConvNet (70.3%), FBCSP baseline (67.4%) and several other networks. Stieger *et al*. [35] previously examined the Shallow ConvNet [18], on the Med-62 dataset and reported that DL-based decoders can outperform online performance, and that using all of the available electrodes provides additional benefit to using just the electrodes around the sensorimotor cortex. The classification accuracy of ShallowConvNet can reach near 79% on session 3 of Med-62 dataset. This cannot be directly compared to our results, since we are using different training and test set and different data clipping strategies. These works, along with several others, show the promise of DL-based decoders for MI-BCI. However, while there are several successful DL models that have already been proposed for BCI, and more are certainly being developed, it is still unclear which, if any, of these models perform the best in a general setting.

Previous works have also compared different basic generic deep learning architectures on motor imagery tasks [36], [37]. These results may provide some guidance for improving DL-based BCI decoders, but still do not look at state of the art models. Here, we aim to address this by comparing multiple state-of-the-art deep learning networks that were specifically designed for BCI motor imagery tasks. From the experiment results, we have shown that EEGNet has the best performance on one dataset under these selected conditions among the models we have investigated and outperforms online accuracy on average as well. The depth of the network does not seem to be the most important factor in predicting a model’s performance. Though the models we tested have varying depths, they all performed similarly on the MBT-42 dataset. In addition, though EEGNet and Shallow ConvNet have similar depths, EEGNet outperforms the other models on the Med-62 datasets. The way to deal with inputs from different channels are also similar among these three models, since they all applied a depthwise convolutional layer to mix data from all channels in the early part of models. A possible reason for the high performance of EEGNet on Med-62 dataset might come from their special design of separable convolutional layers, which may potentially be capable of extracting more strong features related to this task. The Shallow and Deep ConvNets had the lowest training cost which may be an important factor for performing offline data analysis on lowered powered machines or in a time sensitive environment.

### A. The Role of Preprocessing

**Fig. 6.**
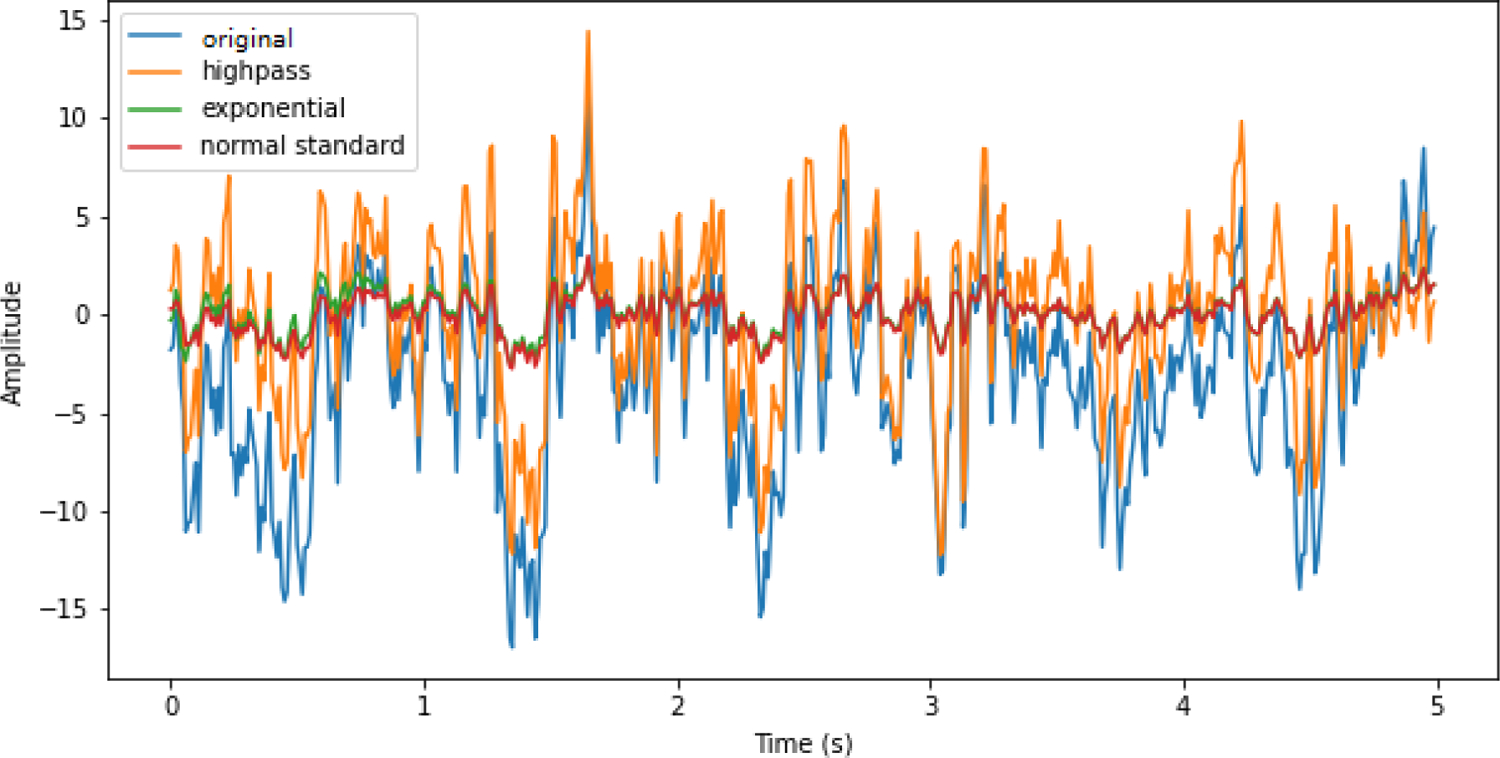
Example of different preprocessing methods on a data snippet. The scale of highpass-filtered data with a cutoff frequency of 1 Hz is almost the same as original data. The scale of data after exponential moving standardization and normal standardization is similar to each other, far smaller than original data, which may benefit deep model training.

The performance of deep learning model is greatly influenced by its input scale. Without data scaling, a deep learning model will learn larger weight parameters, which can cause instability and undermine the performance [38]. Figure 6 shows an illustration of different preprocessing methods on a real data snippet of 5 seconds length from Med-62 dataset. Here the term ‘original data’ refers to the data we directly obtained from the released dataset, which has already been minimally preprocessed by the authors. The orange line is the signal after going through a highpass filter with a cutoff frequency of 1 Hz. The data scale of this highpass-filtered data is almost the same as the original data, which is the blue line, since it only removes the low frequency component of the signal. On the other hand, exponential moving standardization (green line) and the normal standardization (red line) can rescale the data to a range which is suitable for model training, and the preprocessed data using these two methods are almost the same. From Figure 4, the accuracy improvement from preprocessing is much higher in Med-62 dataset. We checked the average standard deviation of original EEG recordings of two datasets, which is a rough reflection of input scale. The standard deviation averaged over all subjects is 28.2 in MBT-42 dataset, and 1335.0 in Med-62 dataset. Although there is not a clear threshold to discriminate ‘normal scale’ and ‘abnormal scale’, this indicates that Med-62 dataset is far from normal distribution, so that it can benefit more from preprocessing. The MB3D model is the one which was affected the least by input scale. One possible explanation for this may be its multi-branch structure. If one branch died out because of abnormal inputs and its subsequent consequence like gradient vanishing, the other branches still reserve the chance to fit well so that the model can still classify correctly.

### B. Subject-Wise Clustering

**Fig. 7.**
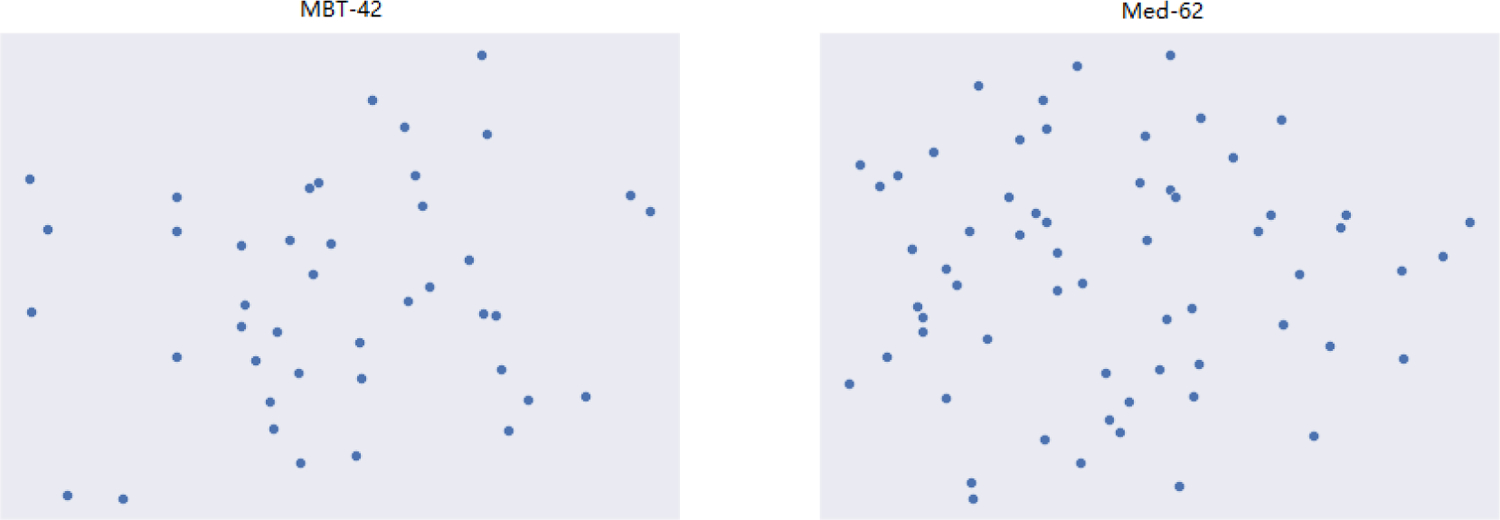
Visualization of zero-centered accuracy vectors on two datasets using t-SNE. No clear subject clusters can be found.

In addition to comparing the overall performances of the various deep learning architectures, we also wanted to explore subject-specific performance between the models. Although some models reached higher accuracies than others on average, it could be the case that specific models work well for some subjects and not others. To test if this is the case, we plotted the subjects’ performance in a five-dimensional space where the accuracy from each model is a single dimension. Since the differences in average accuracy between subjects was much larger than any differences between models for a single subject, we needed to center the subjects’ accuracies in order to focus on any model-specific differences. To remove the effects of the subject’s average performance, we zero-centered their accuracies: (**x**−*mean*(***x***) is the zero-centered vector of **x**). Here, subjects that performed similarly among the various models are closer together in space (ex. if subject A and B both performed better with Shallow Net than with EEGNet they would be closer together). Using t-distributed stochastic neighbor embedding (t-SNE) [39], [40], a statistical method for visualizing high-dimensional data, this five-dimensional space can be visualized in two-dimensions, shown in Figure 7.

Deep learning models with different connective structures might have different preference on special EEG features of certain subjects. In other words, there might exist a kind of phenomenon that: within a subset of subjects with a common EEG feature, or MI strategy, a group of deep learning models perform better, while within another subset of subjects with a different EEG feature, another group of deep learning models perform better. This difference might be expected due to the differences in architectures of the models, and the abilities of the various layers to extract different features. The t-SNE plots in Figure 7 explore how similarly subjects perform among the different models. Here, distinct groups of subjects, or clusters, would signal that there are sub-populations of subjects with unique features that result in them reaching higher performance with some models rather than others. The results of Figure 7 show that subjects are relatively spread out across this space. This suggests that if the models are extracting different features, then these features are relatively distributed across different subjects. Future work could further explore this idea by comparing the electrophysiology between subjects that are the furthest away from each other in this space to extract the features causing the difference in accuracies between models. Including different types of models in this analysis, such as RNNs, may also yield even more stark differences as the architectures extract different features from the data.

Since the clusters are not clear from the two plots in Figure 7, this kind of model preference is weak based on our experiment result. However, we believe it’s still a good direction to explore if we can have more data samples and more models to compare in the future.

### C. Future View of Deep Learning

Deep learning has shown success on improving the power of BCI systems. We believe deep learning has further potential on evolving BCI systems. EEG is one of the most commonly used non-invasive BCI inputs, which has low signal-noise ratio (SNR). As a result, extracting features from such a noisy signal is harder for traditional methods in processing EEG signals. As mentioned, the feature extraction step requires lots of manual work and prior knowledge, and may cause information loss. From deep learning, we can have a better option to automatically generate useful features from noisy data.

There are still issues to be addressed to drive deep learning further to the online system. First is how to deal with different format of data. Compared to the easy data collection process in most of the computer science fields due to the prosperity of Internet, the human EEG data collection is much more harder because of the tedious routines, time-consuming process of recruiting subjects and conducting experiments. EEG data are collected from different research groups using different systems. Given this, building a unified model which can be trained on various types of data and serve for multiple purposes, can increase the utilization rate of limited EEG data and may become an important issue in the future. The ideal case is that a user can start using BCI system without any pre-training/calibration trials. Through the early stage of interacting, the DL model can perform gradual adjustment along uses by the user.

Most of the current models treat each trial equally, which means we can arbitrarily shuffle the order of trials and sessions. However, in real scenario, a subject may generate gradually varied features during BCI training, which might be an additional challenge for decoding. We believe that future investigations can be extended to look into effects of training of MI-BCI using deep learning.

In our work, the performance distribution among subjects in two datasets are not identical. The distribution in MBT-42 dataset is like pear-shaped, while that in Med-62 dataset is much like a spindle. In this case, we can find that different dataset may include different proportion of ‘poor performers’, ‘moderate performers’, and ‘good performers’. Future investigation should be extended to examine effects of deep learning on various performance sub-groups.

## Supplementary Material

supp1-3198041

## Figures and Tables

**TABLE I:** Accuracies of Deep Learning Models and Online Experiments Are Given in Percentage. Stars Denotes the Significant Greater Performance Over Online Results (P Values From Wilcoxon Signed-Rank Test, **: P<0.01). All P Values Have Gone Through Adjustment of False-Discovery-Rate

	EEGNet	ShallowConv	DeepConv	MB3D	ParaAtt	Online
MBT-42	73.65±16.1	71.79±16.9	71.90±16.1	73.12±16.7	72.45±16.7	70.90±16.0
Med-62	75.47±10.8**	72.21±11.8	72.74±11.1	72.66±12.2	73.09±11.9	68.10±12.6

**TABLE II:** Average Model Training Time per Subject of Deep Learning Models on Two Datasets in Seconds

	EEGNet	ShallowConv	DeepConv	MB3D	ParaAtt
MBT-42	3.336	2.621	2.768	17.896	4.457
Med-62	3.990	3.076	3.051	20.037	5.196

**TABLE III:** Average Model Inference Time of Deep Learning Models for a Batch of Samples and One Single Sample in Milliseconds. All Input Samples Are Fixed to Time Length of 300

	EEGNet	ShallowConv	DeepConv	MB3D	ParaAtt
125 samples	16.4	20.1	21.2	117.3	28.1
1 sample	1.8	1.6	1.9	15.4	2.4

## References

[1] He B, Yuan H, Meng J, and Gao S, “Brain–computer interfaces,” in Neural Engineering. Cham, Switzerland: Springer, 2020, pp. 131–183.

[2] Wolpaw JR and McFarland DJ, “Control of a two-dimensional movement signal by a noninvasive brain-computer interface in humans,” Proc. Nat. Acad. Sci. USA, vol. 101, no. 51, pp. 17849–17854, Dec. 2004. 15585584 10.1073/pnas.0403504101 PMC535103

[3] Purtscheller G and Neuper C, “Motor imagery and direct brain-computer communication,” Proc. IEEE, vol. 89, no. 7, pp. 1123–1134, Jul. 2001.

[4] Yuan H and He B, “Brain–computer interfaces using sensorimotor rhythms: Current state and future perspectives,” IEEE Trans. Biomed. Eng, vol. 61, no. 5, pp. 1425–1435, May 2014. 24759276 10.1109/TBME.2014.2312397 PMC4082720

[5] He B, Baxter B, Edelman BJ, Cline CC, and Ye WW, “Noninvasive brain-computer interfaces based on sensorimotor rhythms,” Proc. IEEE, vol. 103, no. 6, pp. 907–925, Jun. 2015. 10.1109/jproc.2015.2407272 PMC8323842 34334804

[6] Edelman BJ , “Noninvasive neuroimaging enhances continuous neural tracking for robotic device control,” Sci. Robot, vol. 4, no. 31, Jun. 2019, Art. no. eaaw6844. 10.1126/scirobotics.aaw6844 PMC6814169 31656937

[7] de Vries S and Mulder T, “Motor imagery and stroke rehabilitation: A critical discussion,” J. Rehabil. Med, vol. 39, no. 1, pp. 5–13, 2007. 17225031 10.2340/16501977-0020

[8] Yuan H, Liu T, Szarkowski R, Rios C, Ashe J, and He B, “An EEG and fMRI study of motor imagery: Negative correlation of bold and EEG activity in primary motor cortex,” NeuroImage, vol. 49, pp. 2596–2606, Jan. 2010. 19850134 10.1016/j.neuroimage.2009.10.028 PMC2818527

[9] LaFleur K, Cassady K, Doud A, Shades K, Rogin E, and He B, “Quadcopter control in three-dimensional space using a noninvasive motor imagery-based brain–computer interface,” J. Neural Eng, vol. 10, no. 4, Aug. 2013, Art. no. 046003. 10.1088/1741-2560/10/4/046003 PMC3839680 23735712

[10] Meng J, Zhang S, Bekyo A, Olsoe J, Baxter B, and He B, “Noninvasive electroencephalogram based control of a robotic arm for reach and grasp tasks,” Sci. Rep, vol. 6, no. 1, pp. 1–15, Dec. 2016. 27966546 10.1038/srep38565 PMC5155290

[11] Mokienko OA, Lyudmila CA, Frolov AA, and Bobrov PD, “Motor imagery and its practical application,” Neurosci. Behav. Physiol, vol. 44, pp. 483–489, Jun. 2014.

[12] Wang T, Deng J, and He B, “Classifying EEG-based motor imagery tasks by means of time–frequency synthesized spatial patterns,” Clin. Neurophysiol, vol. 115, no. 12, pp. 2744–2753, Dec. 2004. 15546783 10.1016/j.clinph.2004.06.022

[13] Blankertz B, Tomioka R, Lemm S, Kawanabe M, and Müller KR, “Optimizing spatial filters for robust EEG single-trial analysis,” IEEE Signal Process. Mag, vol. 25, no. 1, pp. 41–56, Jan. 2008.

[14] Ramoser H, Müller-Gerking J, and Pfurtscheller G, “Optimal spatial filtering of single trial EEG during imagined hand movement,” IEEE Trans. Neural Syst. Rehabil. Eng, vol. 8, no. 4, pp. 441–446, Dec. 2000. 10.1109/86.895946 11204034

[15] Ang KK, Chin ZY, Zhang H, and Guan C, “Filter bank common spatial pattern (FBCSP) in brain-computer interface,” in Proc. IEEE Int. Joint Conf. Neural Netw. IEEE World Congr. Comput. Intell, Jun. 2008, pp. 2390–2397.

[16] Lotte F , “A review of classification algorithms for EEG-based brain–computer interfaces: A 10 year update,” J. Neural Eng, vol. 15, no. 3, Jun. 2018, Art. no. 031005. 10.1088/1741-2552/aab2f2 29488902

[17] Craik A, He Y, and Contreras-Vidal JL, “Deep learning for electroencephalogram (EEG) classification tasks: A review,” J. Neural Eng, vol. 16, no. 3, Jun. 2019, Art. no. 031001. 10.1088/1741-2552/ab0ab5 30808014

[18] Schirrmeister RT , “Deep learning with convolutional neural networks for EEG decoding and visualization,” Hum. Brain Mapping, vol. 38, pp. 5391–5420, Nov. 2017. 10.1002/hbm.23730 PMC5655781 28782865

[19] Sakhavi S, Guan C, and Yan S, “Learning temporal information for brain-computer interface using convolutional neural networks,” IEEE Trans. Neural Netw. Learn. Syst, vol. 29, no. 11, pp. 5619–5629, Nov. 2018. 29994075 10.1109/TNNLS.2018.2789927

[20] Lawhern VJ, Solon AJ, Waytowich NR, Gordon SM, Hung CP, and Lance BJ, “EEGNet: A compact convolutional neural network for EEG-based brain–computer interfaces,” J. Neural Eng, vol. 15, no. 5, Oct. 2018, Art. no. 056013. 10.1088/1741-2552/aace8c 29932424

[21] Tang Z, Li C, and Sun S, “Single-trial EEG classification of motor imagery using deep convolutional neural networks,” Optik, vol. 130, pp. 11–18, Feb. 2017.

[22] Dose H, Møller JS, Iversen HK, and Puthusserypady S, “An end-toend deep learning approach to MI-EEG signal classification for BCIs,” Expert Syst. Appl, vol. 114, pp. 532–542, Dec. 2018.

[23] Jingwei L, Yin C, and Weidong Z, “Deep learning EEG response representation for brain computer interface,” in Proc. 34th Chin. Control Conf. (CCC), Jul. 2015, pp. 3518–3523.

[24] Liu X, Shen Y, Liu J, Yang J, Xiong P, and Lin F, “Parallel spatial–temporal self-attention CNN-based motor imagery classification for BCI,” Frontiers Neurosci, vol. 14, Dec. 2020, Art. no. 587520. 10.3389/fnins.2020.587520 PMC7759669 33362458

[25] Tabar YR and Halici U, “A novel deep learning approach for classification of EEG motor imagery signals,” J. Neural Eng, vol. 14, no. 1, 2017, Art. no. 016003. 10.1088/1741-2560/14/1/016003 27900952

[26] Amin SU, Alsulaiman M, Muhammad G, Mekhtiche MA, and Hossain MS, “Deep learning for EEG motor imagery classification based on multi-layer CNNs feature fusion,” Future Gener. Comput. Syst, vol. 101, pp. 542–554, Dec. 2019.

[27] Bashivan P, Rish I, Yeasin M, and Codella N, “Learning representations from EEG with deep recurrent-convolutional neural networks,” 2015, arXiv:1511.06448.

[28] Zhao X, Zhang H, Zhu G, You F, Kuang S, and Sun L, “A multi-branch 3D convolutional neural network for EEG-based motor imagery classification,” IEEE Trans. Neural Syst. Rehabil. Eng, vol. 27, no. 10, pp. 2164–2177, Oct. 2019. 31478864 10.1109/TNSRE.2019.2938295

[29] Tangermann M , “Review of the BCI competition IV,” Frontiers Neurosci, vol. 6, no. 1, p. 55, 2012. 10.3389/fnins.2012.00055 PMC3396284 22811657

[30] Meng J and He B, “Exploring training effect in 42 human subjects using a non-invasive sensorimotor rhythm based online BCI,” Frontiers Hum. Neurosci, vol. 13, p. 128, Apr. 2019. 10.3389/fnhum.2019.00128 PMC6481252 31057380

[31] Stieger JR, Engel SA, and He B, “Continuous sensorimotor rhythm based brain computer interface learning in a large population,” Sci. Data, vol. 8, no. 1, pp. 1–10, Dec. 2021. 33795705 10.1038/s41597-021-00883-1 PMC8016873

[32] Stieger JR, Engel S, Jiang H, Cline CC, Kreitzer MJ, and He B, “Mindfulness improves brain–computer interface performance by increasing control over neural activity in the alpha band,” Cerebral Cortex, vol. 31, no. 1, pp. 426–438, Jan. 2021. 32965471 10.1093/cercor/bhaa234 PMC7727383

[33] Vaswani A , “Attention is all you need,” in Proc. Adv. Neural Inf. Process. Syst, 2017, pp. 5998–6008.

[34] Towns J , “XSEDE: Accelerating scientific discovery,” Comput. Sci. Eng, vol. 16, no. 5, pp. 62–74, 2014.

[35] Stieger JR, Engel SA, Suma D, and He B, “Benefits of deep learning classification of continuous noninvasive brain–computer interface control,” J. Neural Eng, vol. 18, no. 4, 2021, Art. no. 046082. 10.1088/1741-2552/ac0584 PMC9305984 34038873

[36] Hernández LG and Antelis JM, “A comparison of deep neural network algorithms for recognition of EEG motor imagery signals,” in Proc. Mex. Conf. Pattern Recognit Cham, Switzerland: Springer, 2018, pp. 126–134.

[37] León J , “Deep learning for EEG-based motor imagery classification: Accuracy-cost trade-off,” PLoS ONE, vol. 15, no. 6, Jun. 2020, Art. no. e0234178. 10.1371/journal.pone.0234178 PMC7289369 32525885

[38] Bishop CM , Neural Networks for Pattern Recognition. London, U.K.: Oxford Univ. Press, 1995.

[39] Pedregosa F , “Scikit-learn: Machine learning in Python,” J. Mach. Learn. Res, vol. 12, pp. 2825–2830, Jan. 2012.

[40] Van der Maaten L and Hinton G, “Visualizing data using t-SNE,”J. Mach. Learn. Res, vol. 9, no. 11, pp. 2579–2605, 2008.

