# Supplementary material for: On the Deep Learning Models for EEG-Based Brain-Computer Interface Using Motor Imagery
Source: IEEE Trans Neural Syst Rehabil Eng. Author manuscript; Available in PMC 2022 Aug 28. (PMC9420068; doi:10.1109/TNSRE.2022.3198041)
Supplement: supp1-3198041 [file NIHMS1831290-supplement-supp1-3198041.pdf]

Supplementary Information

Of

On The Deep Learning Models for EEG-based Brain-Computer Interface Using Motor Imagery

Hao Zhu, Dylan Forenzo, Bin He

Supplementary Note: DETAILED PARAMETERS OF DEEP LEARNING MODELS

All model performances shown in the main paper have already been fine-tuned and selected the best results among different hyper-parameters.For each model, we tested its performances on 5 learning rates (0.001, 0.005, 0.01, 0.05, 0.1) and 3 weight decay rates (0, 0.001, 0.01). The full result is shown in Supplementary Figure S1.

For EEGNet, we used exactly the same model architecture as EEGNet-8,2 model proposed in the original work. We fit the model using the Adam optimizer with learning rate=0.01 in MBT-42, 0.005 in Med-62. Weight decay rates are both zero.

For deep ConvNet, we used the same model architecture as in the original paper, except for the convolution kernel length in temporal dimension. In the original work, the kernel length of four convolution blocks are (10, 10, 10, 10), which we changed to (8, 8, 8, 4). This is because of the different sampling rates of datasets used in ConvNet and our work (250 Hz v.s. 100/128 Hz), which makes our data snippet shorter than that in the ConvNet work. If we keep the original kernel length, the total receptive field will exceed the whole length of our data. We fit the model using the Adam optimizer with learning rate=0.01 in MBT-42, 0.005 in Med-62. Weight decay rates are both 0.001.

For shallow ConvNet, we used the exactly the same model architecture as in the original paper. We fit the model using the Adam optimizer with learning rate=0.1 in both datasets. The weight decay rate is 0.001 in MBT-42, 0.01 in Med-62.

For ParaAtt, we used exactly the same model architecture as in the original paper. We fit the model using the Adam optimizer with learning rate=0.1 and weight decay rate=0.01 in both datasets.

For the last model, MB3D, we used the same model structure except for the input tensor. In the original work, they embedded a total number of 22 EEG channels into a 6×7 matrix. In our work, we embedded a total number of 59 channels into a 9×9 matrix, arranged as Supplementary Table S1. We fit the model using the Adam optimizer with learning rate=0.001 in MBT-42, 0.005 in Med-62. Weight decay rates are both zero.

For the first four models except for MB3D, we trained 30 epochs for each subject, while the epoch number is 15 in MB3D. The main reason is that the training time of MB3D is too long compared to other models. The improvement is also limited if we train 15 more epochs for this model. For all the models, we used the batch size of 32 and use the epoch with the best performance on validation set for testing. We illustrate the detailed model structures and parameters in Supplementary Figure S2. You can also reach out to the original works for more information.

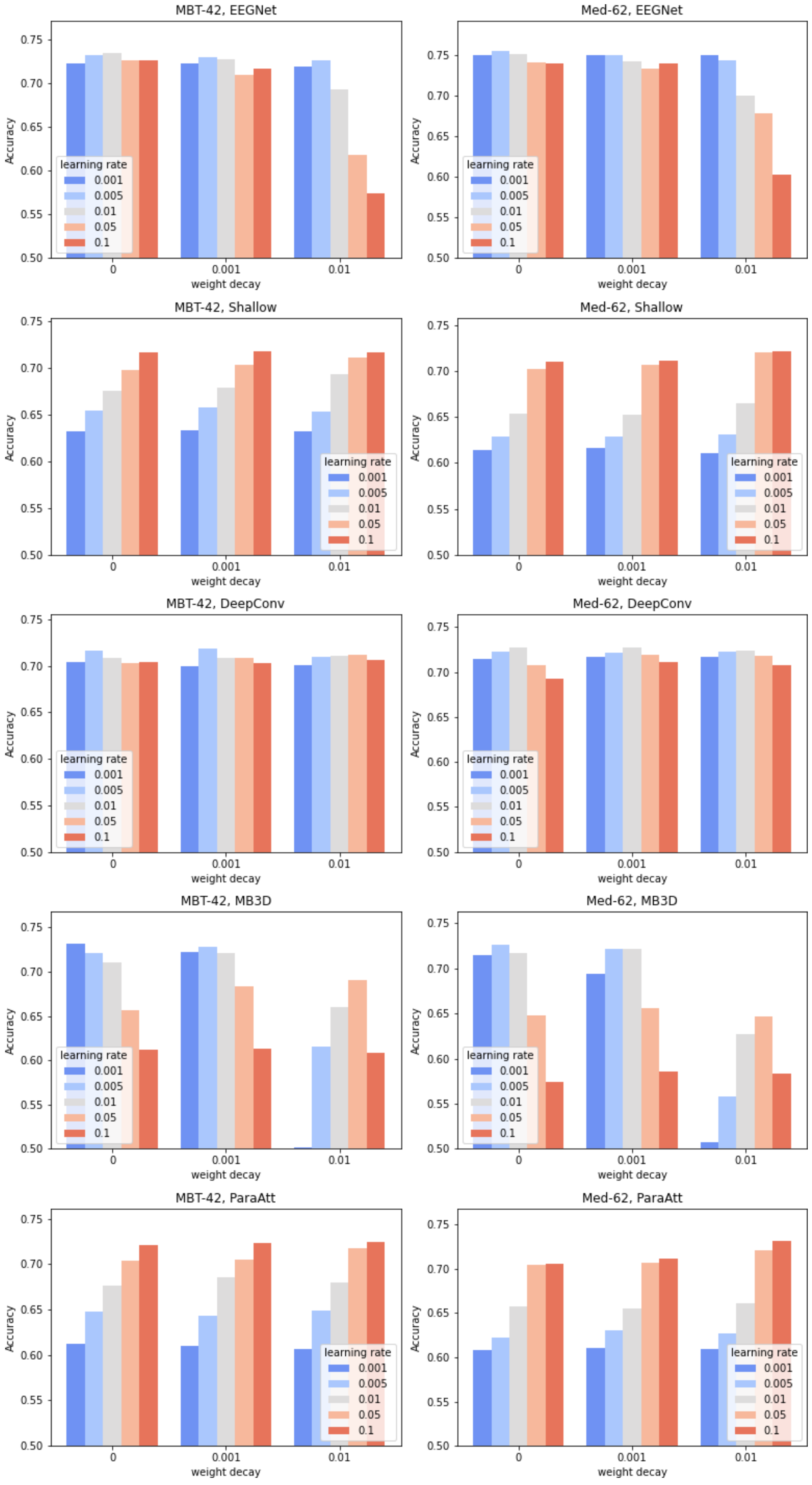

Supplementary Fig. S1. Model accuracy comparison within 5 learning rates and 3 weight decay rates.

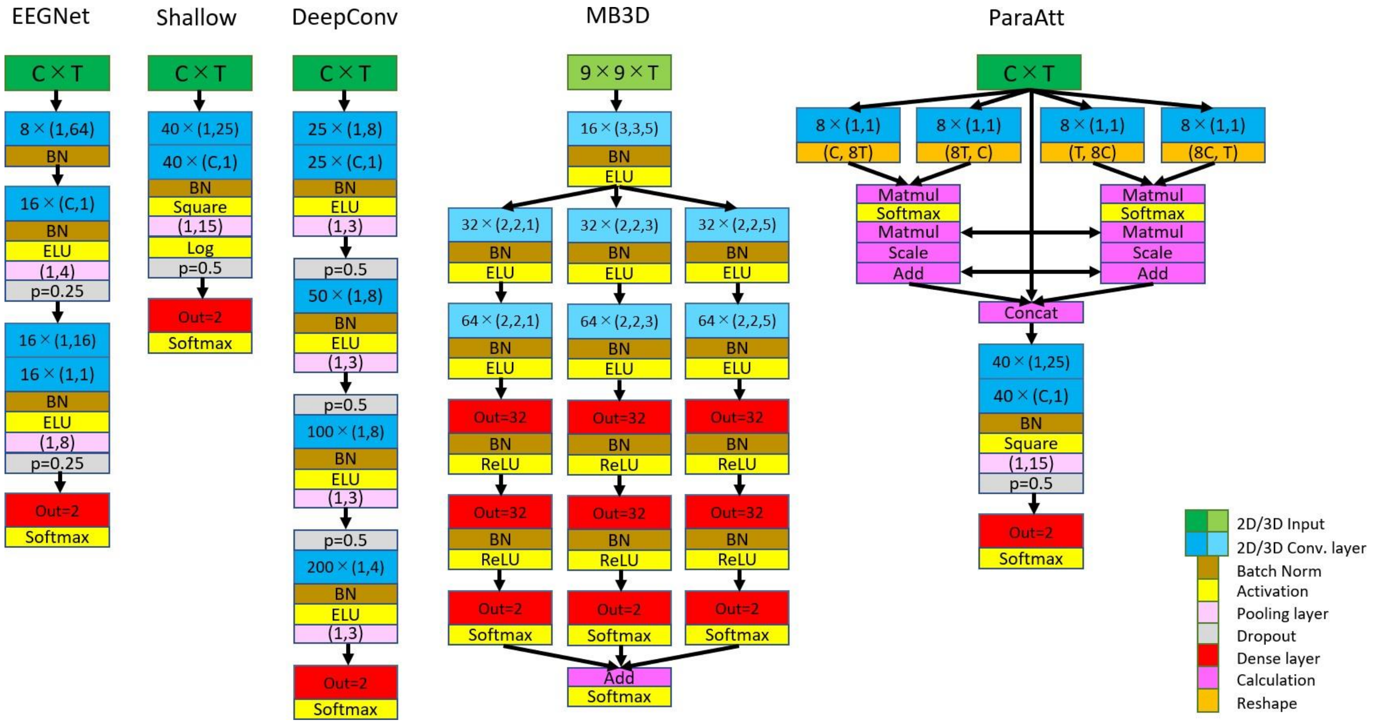

Supplementary Fig. S2. Brief illustration of each model structure. The parameters in convolution blocks denote kernel number×kernel size. The parameters in pooling blocks denote the stride of the pooling layers. The dropout rates are shown in corresponding dropout blocks. The parameters in dense blocks denote their output hidden unit numbers.

Supplementary TABLE S1

Channel arrangement for the $9 \times 9 \times T$ input tensor of MB3D model.

0	0	FP1	0	FPZ	0	FP2	0	0
0	0	AF3	0	AFZ	0	AF4	0	0
F7	F5	F3	F1	FZ	F2	F4	F6	F8
FT7	FC5	FC3	FC1	FCZ	FC2	FC4	FC6	FT8
T7	C5	C3	C1	CZ	C2	C4	C6	T8
TP7	CP5	CP3	CP1	CPZ	CP2	CP4	CP6	TP8
P7	P5	P3	P1	PZ	P2	P4	P6	P8
0	PO7	PO3	0	POZ	0	PO4	PO8	0
0	0	O1	0	OZ	0	O2	0	0

Supplementary TABLE S2

Detailed accuracy results of MBT-42 dataset.

Subject	EEGNet	Shallow	DeepConv	MB3D	ParaAtt	Online
1	0.688	0.824	0.720	0.816	0.776	0.488
2	0.544	0.536	0.552	0.528	0.488	0.516
3	0.960	0.912	0.920	0.928	0.912	0.956
4	0.528	0.504	0.552	0.592	0.600	0.532
5	0.864	0.864	0.864	0.848	0.832	0.776
6	0.848	0.800	0.816	0.832	0.792	0.564
7	0.544	0.560	0.528	0.592	0.520	0.644
8	0.904	0.936	0.912	0.888	0.952	0.476
9	0.768	0.752	0.832	0.864	0.728	0.484
10	0.856	0.848	0.848	0.872	0.856	0.908
11	0.632	0.648	0.680	0.584	0.624	0.572
12	0.496	0.568	0.544	0.520	0.528	0.644
13	0.936	0.904	0.864	0.936	0.944	0.756
14	0.968	0.976	0.952	0.984	0.976	0.780
15	0.984	0.992	0.952	0.992	0.984	0.548
16	0.664	0.752	0.736	0.640	0.816	0.724
17	0.950	0.950	0.942	0.942	0.983	0.800
18	0.658	0.717	0.700	0.742	0.708	0.925
19	0.917	0.892	0.908	0.917	0.867	0.808
20	0.975	0.967	0.942	0.958	0.942	0.967
21	0.625	0.592	0.608	0.633	0.642	0.917
22	0.658	0.725	0.675	0.600	0.708	0.713
23	0.975	0.917	0.850	0.800	0.908	0.967
24	0.700	0.667	0.767	0.758	0.717	0.450
25	0.883	0.842	0.900	0.900	0.875	0.892
26	0.925	0.875	0.917	0.892	0.908	0.675
27	0.958	0.958	0.933	0.975	0.967	0.904
28	0.783	0.733	0.758	0.750	0.767	0.583
29	0.633	0.533	0.483	0.483	0.525	0.617
30	0.758	0.700	0.683	0.700	0.683	0.908
31	0.583	0.483	0.542	0.500	0.592	0.525
32	0.758	0.758	0.742	0.742	0.767	0.708
33	0.617	0.500	0.542	0.550	0.533	0.750
34	0.650	0.575	0.608	0.633	0.600	0.758
35	0.692	0.542	0.600	0.633	0.592	0.658
36	0.550	0.525	0.558	0.517	0.550	0.750
37	0.475	0.458	0.450	0.475	0.450	0.525
38	0.542	0.600	0.550	0.617	0.575	0.500
39	0.567	0.533	0.542	0.592	0.542	0.900
40	0.717	0.617	0.667	0.742	0.667	0.900
41	0.592	0.483	0.550	0.575	0.550	0.679
42	0.608	0.633	0.508	0.667	0.483	0.633

Supplementary TABLE S3

DETAILED ACCURACY RESULTS OF MED-62 DATASET.

Subject	EEGNet	Shallow	DeepConv	MB3D	ParaAtt	Online
1	0.653	0.587	0.553	0.553	0.567	0.713
2	0.927	0.847	0.860	0.913	0.873	0.760
3	0.853	0.847	0.820	0.820	0.853	0.567
4	0.493	0.507	0.493	0.400	0.427	0.693
5	0.713	0.707	0.720	0.727	0.733	0.913
6	0.527	0.467	0.573	0.493	0.587	0.507
7	0.840	0.813	0.853	0.807	0.780	0.560
8	0.720	0.733	0.673	0.727	0.753	0.767
9	0.807	0.820	0.787	0.847	0.840	0.793
10	0.700	0.613	0.680	0.647	0.660	0.640
11	0.773	0.640	0.647	0.640	0.713	0.700
12	0.813	0.793	0.813	0.793	0.827	0.540
13	0.787	0.793	0.773	0.773	0.780	0.600
14	0.673	0.607	0.660	0.647	0.533	0.533
15	0.753	0.580	0.633	0.687	0.607	0.713
16	0.780	0.740	0.793	0.687	0.767	0.527
17	0.740	0.740	0.680	0.820	0.787	0.560
18	0.593	0.567	0.580	0.607	0.573	0.580
19	0.787	0.693	0.660	0.547	0.627	0.793
20	0.920	0.953	0.967	0.947	0.953	0.773
21	0.733	0.660	0.633	0.673	0.647	0.487
22	0.867	0.867	0.887	0.867	0.880	0.600
23	0.773	0.773	0.760	0.800	0.780	0.873
24	0.913	0.840	0.807	0.780	0.853	0.607
25	0.753	0.733	0.707	0.800	0.720	0.760
26	0.767	0.713	0.740	0.713	0.713	0.720
27	0.980	0.973	0.960	0.980	0.973	0.520
28	0.700	0.687	0.713	0.720	0.660	0.900
29	0.767	0.760	0.727	0.713	0.753	0.853
30	0.747	0.733	0.793	0.740	0.773	0.713
31	0.527	0.513	0.540	0.513	0.513	0.500
32	0.887	0.887	0.880	0.893	0.920	0.580
33	0.740	0.620	0.787	0.587	0.707	0.627
34	0.773	0.727	0.700	0.733	0.760	0.653
35	0.713	0.680	0.747	0.760	0.640	0.707
36	0.900	0.907	0.907	0.933	0.893	0.860
37	0.880	0.893	0.880	0.827	0.913	0.600
38	0.553	0.547	0.500	0.567	0.533	0.740
39	0.847	0.707	0.760	0.840	0.713	0.960
40	0.740	0.820	0.767	0.753	0.767	0.500
41	0.660	0.660	0.673	0.680	0.660	0.733
42	0.747	0.740	0.727	0.753	0.713	0.580
43	0.927	0.913	0.853	0.893	0.920	0.787
44	0.827	0.680	0.720	0.727	0.687	0.727
45	0.673	0.627	0.687	0.620	0.613	0.760
46	0.787	0.813	0.827	0.793	0.787	0.707
47	0.687	0.660	0.620	0.600	0.640	0.573
48	0.640	0.600	0.527	0.587	0.580	0.473
49	0.707	0.720	0.720	0.680	0.747	0.760
50	0.793	0.720	0.747	0.687	0.733	0.853
51	0.753	0.693	0.720	0.727	0.693	0.620
52	0.740	0.747	0.753	0.800	0.767	0.880
53	0.847	0.853	0.793	0.820	0.867	0.780
54	0.580	0.500	0.547	0.527	0.560	0.607
55	0.680	0.680	0.667	0.660	0.707	0.573
56	0.707	0.707	0.720	0.700	0.740	0.627
57	0.967	0.933	0.940	0.933	0.933	0.880
58	0.793	0.793	0.720	0.787	0.793	0.627
59	0.733	0.673	0.640	0.647	0.707	0.713
60	0.767	0.740	0.787	0.773	0.767	0.860
61	0.567	0.553	0.587	0.580	0.620	0.600
62	0.800	0.680	0.713	0.800	0.733	0.513
